# Food Texture Design by 3D Printing: A Review

**DOI:** 10.3390/foods10020320

**Published:** 2021-02-03

**Authors:** Tatiana Pereira, Sónia Barroso, Maria M. Gil

**Affiliations:** 1 MARE—Marine and Environmental Sciences Centre, Polytechnic of Leiria, Cetemares, 2520-620 Peniche, Portugal; tatiana.m.pereira@ipleiria.pt (T.P.); sonia.barroso@ipleiria.pt (S.B.); 2 MARE—Marine and Environmental Sciences Centre, School of Tourism and Maritime Technology, Polytechnic of Leiria, Cetemares, 2520-620 Peniche, Portugal

**Keywords:** food design, texture, 3D printing, personalization, structure, model

## Abstract

An important factor in consumers’ acceptability, beyond visual appearance and taste, is food texture. The elderly and people with dysphagia are more likely to present malnourishment due to visually and texturally unappealing food. Three-dimensional Printing is an additive manufacturing technology that can aid the food industry in developing novel and more complex food products and has the potential to produce tailored foods for specific needs. As a technology that builds food products layer by layer, 3D Printing can present a new methodology to design realistic food textures by the precise placement of texturing elements in the food, printing of multi-material products, and design of complex internal structures. This paper intends to review the existing work on 3D food printing and discuss the recent developments concerning food texture design. Advantages and limitations of 3D Printing in the food industry, the material-based printability and model-based texture, and the future trends in 3D Printing, including numerical simulations, incorporation of cooking technology to the printing, and 4D modifications are discussed. Key challenges for the mainstream adoption of 3D Printing are also elaborated on.

## 1. Introduction

The food industry is experiencing a paradigm shift. People’s growing awareness of the food that they consume and the drive for new customized sensory experiences is pushing for the development of new technologies that can satisfy these new consumers’ standards. 

One of these novel technologies, 3D Printing, has been around for a while, however, only in 2007 was it applied for the first time in the production of food structures [1]. Since then, 3D Printing has been used to create visually complex geometrical structures beyond the capabilities of the traditional methods of food production [2,3]. Recently, the focus has shifted from the visual aspects of the products to the control and personalization of the foods’ nutritional characteristics [3,4,5]. The products should not only be visually attractive but also possess a healthy nutritional profile.

3D Printing has also been defined by various other terms, such as Additive Layer Manufacturing (ALM), Solid Freeform Fabrication (SFF), and Rapid Prototyping (RP) [2,6,7]. For simplicity’s sake, 3D Printing is the term used to mention this technology from this point onwards.

This technology has attracted a lot of attention for its versatility and potential application in various production sectors, such as aerospace, electronics, architecture, and medicine [8,9,10]. In the food production sector, this technology has the potential to be used to create personalized food products, enabling the creation of food products with specific design characteristics, flavors and colors, geometric structures, textures, and nutritional profiles [2,5,11,12].

**Figure 1:**
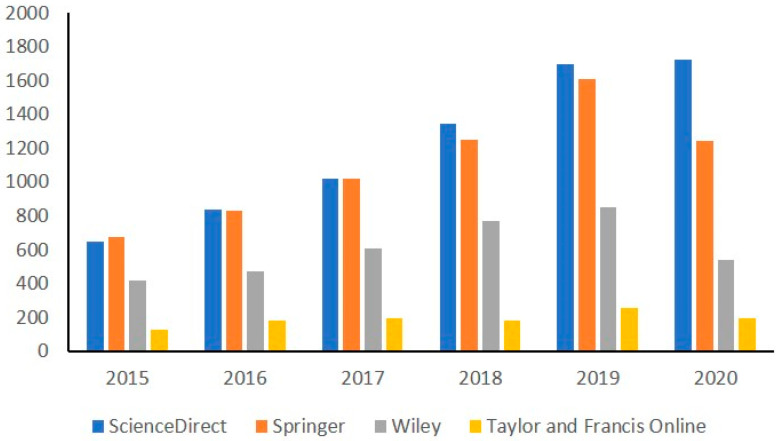
Number of publications returned from a search containing the keywords “(“Additive manufacturing” OR 3D Printing OR three-dimensional printing) AND food” during the period between 2015–2020 (July 2020) in four databases (ScienceDirect, Springer, Wiley, Francis and Taylor Online).

Three-dimensional Printing is an innovative and some say disruptive technology, which is in constant expansion, as seen by the rising number of reviews, book chapters, and research articles published every year. Figure 1 illustrates the number of publications returned from a search containing the keywords (“Additive manufacturing” OR 3D Printing OR three-dimensional printing) AND food from 2015 to 2020, in four databases (ScienceDirect, Springer, Wiley, Francis and Taylor Online). In July 2020, the number of publications in 2020 (in ScienceDirect) was equal to total the numbers of 2019.

Some corporations are already using this technology in the fabrication of their products, such as Hershey’s (chocolates), Barilla (pasta noodle), Ruffles (potato chips), Oreo (cookies), and Mazola (fruits and vegetables) [13]. In the production of meat-based products, Aleph Farms [14] and Meatech [15] use 3D Printing in the production of laboratory-grown meat, and Redefine Meat [16] and Novameat [17] for plant-based meat.

Beyond taste and appearance, one of the major factors in consumer acceptability is the texture and mouthfeel of the food [18,19,20]. While taste and appearance are the factors that attract more attention during food production and consumer purchase, texture is crucial in food preferences and can make a difference at the time of purchase [21]. 

In the case of elderly and people with swallowing problems (dysphagia), their need to consume texture-modified foods, that—for safety reasons—must be pureed, can constitute a problem because of its unappealing look and texture that can lead to food rejection and malnourishment [22,23,24]. Much in the same way, texture can have a big impact on people with food aversions [25]. Another group that can benefit from texture-modifications are people with weight issues (i.e., overweight and obese) who require healthier foods with reduced caloric content [21]. 

Nowadays, there is a growing concern to reduce salt, sugar, and fat contents in foods, however, this task is difficult in the sense that these compounds also play structural and preserving roles on the products [26]. The reduction in and substitution of salt, fat, and sugar can be achieved by using alternative additives, texture modifications, or inhomogeneous spatial dispersion of the compounds [26,27,28]. However, healthier products are often perceived as unappealing and bland when compared with their conventional counterparts [29,30]. 

By using a layered approach, extrusion printing offers a new methodology to produce healthy and attractively textured food by the specific placement of texturing elements in the food [31,32,33].

Focusing on 3D Printing texture design, two distinct research groups, Hemsley et al. [24] and Sungsinchai et al. [34], reviewed the state-of-the-art of 3D Printing technology in the development of food products for people with dysphagia. Hemsley et al. [24] also predicted and listed the future research efforts involved in turning 3D Printing into a mainstream technology. In another publication, Gholamipour-Shirazi et al. [35] reviewed the major findings of over 40 articles using different food materials in terms of the effect of the materials’ rheological properties on the printing outcome, also presenting some of the most significant milestones of the last 11 years of research.

The purpose of this review is to investigate the existing 3D Printing literature and assess the recent developments in the technology concerning food texture design. Section 2 elucidates what texture is and how the food textural properties are evaluated, in addition to presenting the importance of food texture in susceptible populations such as the elderly and people with dysphagia, and in determining the consumers’ level of acceptance. Section 3 provides an overview of 3D Printing, elucidating what it is, which techniques are used in food fabrication, and the advantages and limitations of its use at an industrial level. Section 4 discusses the major findings of the most recent and impactful investigations in material and model-based food texture-modifications with 3D Printing. Section 5 demonstrates the consumers’ perception of 3D printing as a food technology and the acceptability of 3D printed food products. Lastly, Section 6 elaborates on the future trends and the key challenges for the mainstream adoption of 3D Printing.

## 2. Food Texture

While a definitive meaning for texture is still not unanimous, the consensus seems to be, as defined by the International Organization for Standardization (ISO) [36], that texture comprises “all the mechanical, geometrical and surface attributes of a product perceptible by means of mechanical, tactile and, where appropriate, visual and auditory receptors” [18,21,36]. In other words, texture seems to encompass every aspect of the food product that can be perceived by the human senses, particularly by the hands and mouth. 

Both texture perception and preference in humans vary greatly from person to person and are heavily influenced by an individual’s personal experiences and culture [21]. To better illustrate this point, Japan is reported to have over 400 terms to describe food texture, while western countries like the USA and Austria have only 78 and 105, respectively [37].

In susceptible populations, such as the elderly and people with dysphagia, food texture is of utmost importance due to the risk of aspiration and choking [24,34]. This safety hazard demonstrates the need to create food products that follow specific frameworks, such as the one created by the International Dysphagia Diet Standardization Initiative (IDDSI) [38]. This framework categorizes the flow and texture of drinks and foods into an eight-level scale, that can be used by clinics and caregivers during the food’s confection to make it easier to eat [38]. Drinks range from 0–3 as thin (0) and slightly, mildly, and moderately thick (1,2, and 3, respectively), whereas foods are classified between 4–7 (with 8 being regular food) including pureed (4), minced and moist (5), soft and bite-size (6), and regular easy to chew (7) foods [38]. The classification is done by applying several testing methods that use various daily instruments such as syringes (to classify the thickness of liquids (flow test)), spoons (to classify the stickiness of the food (spoon tilt test)), and forks (to classify the hardness of the food (fork/spoon pressure and fork/spoon separation test)) to ensure the conformity with the recommended guidelines [38].

Food textural properties are determinant on consumer acceptance and are often used to predict consumer’s preferences and evaluate the foods’ quality [39]. In consumer acceptance analyses, the evaluation is often made in conjunction with other food attributes, such as taste and aroma, since these three attributes play a role in the preference and liking of the foods [21,25]. Moreover, changes in the perception of any of these features can influence the perception of the others [21,25].

Another important concept to consider is the sensory perception or mouthfeel of the food. The mouthfeel is defined as the feel of the food’s texture during its consumption [18]. Studies have shown that texture and mouthfeel play a significant role in food choice, food intake, and even in satiety [39,40].

Consumers’ sensory perceptions are influenced by the packaging and foods’ textural, visual, and tactile properties, creating expectations for the products and thus, the likeliness of the purchase [41]. Jansson-Boyd and Kobescak [42] showed that consumers’ perception of food healthiness is influenced by visual implicit surface textures. They concluded that sweet products, such as biscuits, are more likely to be purchased if they present a less healthy appearance, being perceived as tastier [42]. It was also found that the packaging design and surface patterns influence the sensory perception of ice-cream, chips, coffee, and chocolate beverages [43,44,45]. The authors showed that surfaces with smooth texture intensify the sweetness perception in ice creams, while rough surfaces enhance the bitterness of the coffee and saltiness in potato chips [43,44,45]. Findings like these presented can be useful in the promotion of healthier food products, by playing with the textural properties of the packaging and the product.

The assessment of a samples’ texture is often done using rheological and texture profile analysis (TPA). However, these analyses only measure the mechanical properties of foods and do not exactly mimic the sensory process of the products in the mouth. Furthermore, since the texture is influenced by personal experiences, its mechanical analysis makes it difficult to predict using instrumental tools [18,19,46,47]. Therefore, despite being both time and economically costly, sensory evaluations are still considered the best approach to assess food quality and consumer acceptability [48]. Nevertheless, instrumental tools present the advantage of being able to put texture into measurable units that can be standardized to ensure quality control during the foods’ development and production [21,49].

As mentioned above, the most used form of instrumental analysis is the rheological analysis, which measures the samples’ deformation behavior and flow, and the texture profile analysis (TPA), or double-compression analysis, which simulates the process of mastication during two cycles of deformation or the first two bites in the human mouth [19,32,48].

The textural properties more commonly studied are hardness, cohesiveness, springiness, adhesiveness, chewiness, and gumminess [9,20,50,51]. Hardness is the force necessary to cause a certain level of deformation and the capacity of the food product to retain its shape. Adhesiveness is linked to the bonding ability of the materials’ components and the force required to break the bonds between the samples’ surface and exterior surfaces that the sample comes in contact with. Cohesiveness is associated with the adhesiveness within the sample itself and the samples’ capability to deform before it ruptures. Springiness is associated with the samples’ elasticity and its capability to restore its original structure after compression. Lastly, gumminess and chewiness are associated with the energy needed to fragment and masticate the food, respectively, with gumminess only applied for semi-solid foods and chewiness for solid foods [34,51,52,53].

Every physical and structural attribute will have an influential role on the perceived texture and mouthfeel of the food products, and thus can be used in the design and manufacturing of healthier products with sensory attributes that encourage people to choose a healthier lifestyle [29].

## 3. 3D Food Printing

Recently, 3D Printing has attracted a lot of attention from food manufacturers and academia for its immense potential to tailor food products to specific necessities and preferences [54,55].

Three-dimensional Printing can be categorized into seven categories, but from those, only extrusion or fused deposit modeling (FDM), inkjet printing, binder jetting, selective sintering, and bio-printing have been used in food production [2,35,54,56,57,58]. The materials used and whether they are present in liquid, powder, or semi-solid form influences the choice of printing technique [35,57]. Extrusion requires the materials to present high viscosity with self-supporting properties, whereas Inkjet requires low viscosity [54,56]. Binder jetting and Selective Sintering, both use powdered materials that are fused by lasers (Selective Laser Sintering), hot-air (Selective Hot-Air Sintering and Melting), or sprayed liquids (Binder Jetting) [54,56].

Three-dimensional Printing applies a layer-by-layer approach to the building of the desired structures. Extrusion 3D printing is the most widely studied and applied in food products. This process involves the use of a hydraulic piston to extrude the food materials through a moving nozzle creating structures in layers [2]. The layers are then fused depending on the properties of the materials which can solidify when cooled or form hydrogels [2,54,58]. Mostly used for decorations, Inkjet deposits low viscosity materials continuously or as droplets through a thermal or a piezoelectric head on a support structure to create surface decorations or fillings [2,56].

In Selective Sintering, powder particles are fused by sintering the materials in a selective pattern by applying lasers or hot air to create the designed structures [54,56]. Once finished, a roller pushes more powder on top of the printed layer, providing the new material for the next layer [54]. This allows the production of multi-material food products without the need to use a support structure [58]. Similarly, Binder Jetting also builds structures through the fusion of powder particle materials, using a roller to provide the next layer. However, the fusion is achieved by the ejection of binder fluids that, when in contact with the powder materials, agglomerates the particles together through crosslinking or dissolution-fusion [2,32,54]. Both technologies have been mostly used in confectionery products, building structures based on sugar, or sugar-based, and chocolate powders [2,58,59].

Finally, Bio-printing is the designation given to printing using cellular material and is mostly applied to build tissue-based structures through extrusion, laser-based, or inkjet printing [2,57,60]. Initially, this method was used to construct organs and tissues for human transplants, but nowadays it is being studied to produce meat-based or meat-mimic (plant-based) products as an alternative to traditional meat [8,61].

**Figure 2:**
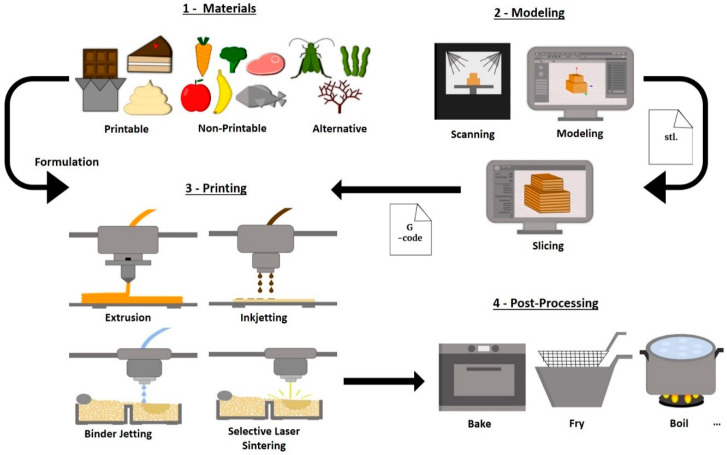
Schematic of the 3D food printing process.

The printing process involves several stages between the choice of material and the printing of the desired structures. First, is the selection of printable material and the development of formulations taking into account the necessary properties for the printing process. Secondly, the model of the desired structure is created using computer-aided software (CAD) or an existing object is scanned to be used as a geometric model. Third, the model is converted into a stereolithography file (stl. file) and sent to a slicing software, where the model layers are created and the printing parameters (speed, temperature, infill, layer height) are set. Finally, a G-code file (a programming language used to give instructions to industrial machine controller computers) with the dimensional and printing instructions is sent to the printer which then builds the desired structure [32,57,61,62,63,64]. Figure 2 schematizes the stages involved in the 3D food printing process, from the choice of material to be used and the modeling and slicing of the 3D structure, to the 3D Printing techniques and the post-processing applied.

3D Printing technology presents a great variety of advantages in the food industry, but the disadvantages still hinder its wider adoption. Table 1 presents a list of the advantages and limitations of using this technology for food printing.

In addition to the above-mentioned advantages of 3D food printing, the use of new components which are not used or are not popular among consumers, such as coproducts and surpluses and by-products of the food processing industry, represents a further benefit. There are significant environmental, nutritional, economic, and social factors that favor the use of processing by-products and surpluses. Such products, being a promising source of valuable substances that possess nutritional properties, are visually unpleasant for consumption, but can be used as printable formulations, following the principles of a circular economy of designing out waste, contributing to the sustainability of the ocean and land. To date, the use of 3D Printing for food has been almost entirely restricted to the production of aesthetic shapes for chocolate and pasta products. However, the potential of this technology should be viewed in a different way, in which food can be tailored to the individual needs, incorporating required nutrients whilst retaining the requirement to also be visually pleasing. Additionally, it is possible to guarantee all the organoleptic characteristics to which consumers are generally familiar with (such as smell, sound, and texture). Moreover, the application of 3D Printing can also promote the consumption of fruits and vegetables and alternative nutrient sources by the general population, through incorporating these between the produced layers, strengthening the food products with the nutrients without their presence being noticed [31].

Despite the many recent advancements in 3D food printing, there remains a myriad of challenges to overcome concerning printable ingredients (the type of food available to print is limited by the printing technique), process (the process is very slow for mass production), and consumers perception (very few people know about food printing and tend to reject it).

Many of the published articles on 3D food printing focus on the search for potential food materials and the optimization of their printing parameters [9,52,68,69,70].

In the review by Gholamipour-Shirazi et al. [35], the authors divided the 3D printed structures and textures in material-based and model-based. In the present review, only the most recent and most significant studies will be further discussed, as a more thorough analysis of older studies can be found elsewhere. Following a similar logic, the discussion of state-of-the-art of 3D printed texture design is divided into material and model-based texture according to the strategies used for the effect. 

## 4. Designing Food Texture with 3D Printing

As mentioned above, 3D Printing can be applied for the creation of novel structures with personalized texture, flavor, and nutritional profile [12,13,54]. It can also be applied to produce novel and intricate geometries that provide unique characteristics to the printed products [11].

The materials’ properties (viscosity, hardness, brittleness), the surface texture, and/or internal structures can influence the perceived mouthfeel of the foods [18,37,71]. The main research in 3D printed food has explored the internal structure of the products due to the limited existing technology capable of manipulating only the food products’ surface.

Three-dimensional Printing can be used to develop healthier food products with reduced fat, sugar, and salt contents [31]. One way that this can be achieved is by component spatial dispersion of the materials [26,27,31]. For example, the dispersion of fat droplets in the foods outer layers can influence the physicochemical properties of the products and create a more pleasant mouthfeel, whereas distributing salt or sugar inhomogeneously throughout food products can enhance their sensory qualities, aiding the production of healthier foods with reduced levels of these components [26,28,29,72]. 

One of the major obstacles to the use of 3D Printing, as previously mentioned, is the lack of abundance of printable materials. Suitable material must present specific characteristics for printing [2,73]. Materials have been divided into three categories: natively printable foods (gels, pastes, dough), non-printable foods (meat, fish, vegetables), and alternative ingredients (insects, algae) [55,57,61].

Several factors can influence the printability of the materials; these involve the formulations and the printing parameters of the materials. The rheological properties of a material, such as flow, viscosity, and yield stress, are used to evaluate and/or predict if the material can be printed by the printer and if it will withhold its shape or collapse upon printing [58,74]. The material needs to present a shear-thinning behavior that provides sufficient flow to be extruded through the nozzle, but enough mechanical strength to uphold its weight and the weight of the top layers [3,31,35,56]. Yield stress evaluates the material’s ability to retain its dimensional shape, while shear storage (G’) and loss modulus (G”) calculations show the viscoelastic properties of the material, and thus its capacity for extrusion [54,75].

During the printing process, several printing parameters (i.e., printing and motor speed, nozzle tip diameter, nozzle height, extrusion rate, temperature) can induce changes in the printed structures [3,11,57]. Hence, there is a need for the optimization of the materials’ formulation and the parameters involved in the printing process, in order to balance the optimal conditions and the desired outcome [52,63,68,70,76].

Some methodologies and guidelines have been proposed to accelerate the evaluation of materials’ printability and the optimization of the food formulations [74,77]. To standardize the measurement of materials’ printability, Kim et al. [77] proposed a printability classification system using hydrocolloids. The authors selected methylcellulose as reference material and used dimensional stability and handling properties to develop a categorized (A, B, C, and D) system for FDM, allowing for the classification of the food printability in comparison to the reference material. Nevertheless, some discrepancies in the deformation rate were observed when comparing the reference material to printed food materials. These differences were justified as being the result of the multi-component composition of the food materials as opposed to the single component of the reference material [77]. To facilitate the formulation and printing process, Zhu et al. [74] studied the correlation between the materials’ rheological properties and the printing performance using tomato puree as a reference, and developed a rational guideline that applies the flow stress as a criterion in the development of food formulations. However, while this guideline can be applied in the development of aqueous-based formulations due to their good correlation with the reference material, the same cannot be said for the formulation of fat-based products, because they were shown to present a very distinct printing behavior from tomato puree [74].

### 4.1. Material-Based Texture

Most materials require flow enhancers to become printable or to enhance their printability [68,78]. For this, the vast majority of studies use hydrocolloids to provide and enhance the viscoelastic attributes necessary for the material to be extruded [58,68,79]. Hydrocolloids increase the viscosity of the formulations allowing their extrusion, in addition to increasing the structural stability and firmness/hardness of the printed products [80,81,82]. However, some attention should be given to the hydrocolloids used and their concentration, because these factors can result in formulations with excessive hardness, hindering the extrusion process and resulting in poor printing accuracy [82]. Tan et al. [79] reviewed the materials and methods used to modify conventional food materials for printing with a focus on texture-modifications suitable for people with dysphagia.

Material composition and the desired mouthfeel influence the choice of hydrocolloids that should be used [21]. Cohen et al. [83] listed the perceived mouthfeel of different concentrations of hydrocolloid composites with their food equivalents to aid in the formulation of food materials for printing, presenting the textural effect that different concentrations could induce on the formulations. 

For the food industry, the bulk of the studies on 3D food printing use extrusion printing, and thus, advancements in knowledge of materials have also been related to those that are suitable for this purpose. As mentioned previously, the materials should present a pseudo-plastic shear-thinning behavior, with enough viscosity to flow through the nozzle but enough elasticity to regain its structure upon printing [56,59,65].

Various material sources have been applied for 3D Printing, with the most used being chocolate, potatoes, and dough [6,7,65,76,84,85]. As alternative material sources, mushrooms, insect flours, and algae have been introduced [9,58,86,87,88].

#### 4.1.1. Source Material Effect on the Printed Product

As mentioned above, the materials’ rheological properties and their formulation influence their printing ability [58]. Nachal et al. [57] reviewed the role of different materials’ properties, such as proteins, fibers, lipids, and carbohydrates in the printing process. The authors mentioned the use of carbohydrates to enhance the materials’ printability and the dimensional stability of the printed products, the physical and sensory property changes caused by the fat content, and the influence of proteins on the texture and microstructure of the printed structures [57].

Great focus is being given to the development of healthy 3D printed fiber-rich food products [4,9,10]. However, the printing of fiber-rich materials, in particular, is a complex affair because of the materials’ characteristics and binding properties, in which the material can clog the nozzle and prevent a continuous extrusion [4,9,10]. High fiber contents are also associated with fragile structures due to particle aggregation. It was found that the addition of semi-skimmed milk and hydrocolloids lessened these problems [10,89]. Nevertheless, the development and printing of fiber-rich snacks from several food materials (mushrooms, composite flours, spinach) has been successful and, at least two of these achieved good acceptability from sensory panels [4,9,10,89].

Enzymes such as transglutaminase have also been applied to enhance the printability of materials used in meat constructs such as surimi, turkey, and scallop puree [90,91]. 

As regards the other 3D printing techniques, fewer studies have been performed using food materials, with only a handful testing the applicability of sugar, chocolate, and cellulose powders in binder jetting printing, and cereal-based materials in selective laser sintering [54,92,93,94].

Three-dimensional Printing is not yet an exact technology, and there are still a lot of gaps in the printing parameters’ effects on the finished product. The printing process presents a major effect on the printed products that can cause changes in their mechanical properties. Several studies have attempted to shorten this gap by correlating the printing parameters to the printed products, however, in the present review, more emphasis will be given to the textural changes, since the mechanical and structural changes have already been elucidated in other reviews [8,11,56].

In terms of texture, Le Tohic et al. [50] proved that the printing process caused significant textural changes in cheese samples. The authors verified that the printed cheese presented lower hardness, thickness, adhesiveness, and higher meltability than untreated cheese, which was explained as being a result of both the heat and shear stress exerted during the printing process that caused structural changes to the fat globules, resulting in softer structures being produced [50]. The nozzle diameter and printing speed also induced textural changes in the hardness and fracturability of rice starch constructs, with larger nozzle diameters producing printed structures with increased hardness [68]. Similarly, Huang et al. [20] evaluated the printing parameters (nozzle diameter), infill levels, and perimeter effect on printing precision and the textural properties of brown rice prints [20]. Better printing precision was achieved using smaller nozzle diameters and wider perimeters [20]. The authors noticed that even though the infill level exerted the greatest effect on the texture of the structure, when increasing the nozzle diameter, the brown rice constructs went from soft textured to harder textures [20].

“Traditional” food materials such as vegetables, fruits, and meat are considered non-printable because of their innate characteristics [55,95]. However, by adding hydrocolloids to enhance printability, it was possible to create healthy and visually attractive structures out of pureed vegetables, fruits, and even fish for children and people with swallowing problems [5,66,96].

Kim et al. [95] developed a standardized preparation method for printing materials by incorporating powdered vegetables into hydrocolloid matrixes which can be adapted for FDM. The authors showed that by using xanthan gum as the hydrocolloid matrix, the printability and rheological properties of the system remained the same, or exhibited reduced differences, regardless of the source of the powder and composition, resulting in prints with good resolution [95]. This study attempted to standardize a methodology to obtain printable materials out of non-printable sources by using hydrocolloids as a matrix. Additionally, the effect of the particle size on the rheology and printing parameters of a spinach powder/xanthan gum dispersion system was investigated [89]. The particle size was shown to increase the hardness and adhesiveness of the system and ensure the dimensional stability of the printed structures. The authors propose the use of particle size to evaluate the materials’ printability and suggested the achievement of different applications by controlling the particle size of the materials [89].

Widely used in the building of scaffolds for prosthetics and organ tissues, the bio-printing of living cell cultures can also create more realistic food textures. The incorporation of lettuce and carrot callus cell tissues in hydrocolloid matrixes and their use as printing materials was proposed to simulate the texture of the actual food [73,97]. Vancauwenberghe et al. [73] applied encapsulated lettuce cells in pectin matrixes, using bovine serum albumin (BSA) to induce porosity. The lettuce/pectin matrixes’ mechanical strength was shown to be positively influenced by the pectin concentration and negatively by the porosity and cell concentration. Pectin concentration affected the cell viability, with higher concentrations hindering the viability [73]. Park et al. [97] incorporated carrot cells in sodium alginate matrixes and cured them using calcium ions. The structures’ dimensionality was shown to be influenced by the cell density, with higher concentrations resulting in prints with lower resolution. The mechanical strength of the carrot/alginate matrix depended on the alginate concentration and cell proliferation. The decrease in the hardness of the structure was justified by the cell growth and the formation of uneven cell clusters [97]. Both studies proved that it is possible to 3D print plant cell tissue with good printability and structural accuracy to simulate the actual food. However, for the development of realistic textures, more research is needed to improve the constructs’ properties [73,97].

The road for printed meat seems to follow a similar path, with companies such as Aleph Farms [14] and Meatech [15] using cultured animal cells as materials to print meat-based products, such as beef and steaks, with the same properties as those from living animals. For plant-based alternatives, Redefine Meat [16] and Novameat [17] used 3D Printing to produce more sustainable meat-free substitutes with the same characteristics as their meat counterparts but with no cholesterol and 95% less environmental impact. The timesaving, environmental sustainability, and slaughter-free potential of this technique represent a great incentive for the adoption of this technology [14,15], but the associated costs are still too high for this to be a viable option for most people [13]. Further studies need to be conducted to meet this demand and make this option feasible for the market.

In the production of printed meat, the simulated texture can be achieved by using multi-materials and the placement of fats in the matrix to simulate the real distribution of these components in a live animal. This was attempted by Dick et al. [98] who recreated a printed lean-beef. The authors created multi-layer models with alternate layers of fat and beef paste and tested the effect of the infill density and fat content on the printed structures after sous vide cooking. Higher infill density enhanced the textural characteristics (hardness and chewiness), while the fat content presented the opposite effect. After cooking, the infill helped with the moisture retention, whereas the fat content induced cooking loss and shrinkage effects on the structure [98]. In another study, the same authors printed pork-paste with a texture suitable for people with dysphagia [78]. It was noticed that, after freezing and heating, the addition of hydrocolloids to the pork-paste decreased the hardness, chewiness, and cohesiveness of the printed meat, making it a potentially suitable product for people with swallowing problems [78]. Nevertheless, the development of printed meat-based structures tailored for people with dysphagia is still slow and there are still big gaps involving formulations and post-processing. Dick et al. [78] refer to the need to develop and optimize food formulations that can keep their textural properties after post-processing and to find post-processing methodologies and conditions that promote particular food characteristics, such as softness and water retention.

Using materials with distinct characteristics to create multi-material layer structures with novel textures has been scarcely applied in other products and not a lot is known about the influence of the multi-material structures on the perceived texture of the printed constructs. Printers with multiple printheads can be used for this purpose, with each nozzle depositing a different material, creating complex multi-material structures with better control on the materials’ distribution and composition [75]. Liu, Zhang, and Yang [75] applied two distinct methodologies on a dual extrusion printer to create multi-material structures using mashed potato and strawberry jam. One methodology consisted of the design of various 3D models followed by the merging of the stl. files, and assignment of distinct parts to an extruder, while the other methodology created a 3D model and assigned different parts of the same model to each extruder. Both methodologies could create visually attractive multi-material structures and create distinct textures within the same products, by interchanging layers with different textures [75].

Multi-material deposition was used to print sesame, chicken, and shrimp paste products and to produce 4D color changing multi-material structures out of potato starch and anthocyanins and pureed purple potatoes and mashed potatoes [99,100,101].

Beyond the material supply used in the printed products, the structure can also influence the perceived texture of the product. In 3D Printing, the creation of distinct textural properties in food constructs is being achieved by introducing pores to the designed model and printing products with different internal structures [62,65,102].

The effects of several parameters, such as the infill patterns and void fraction (porosity), on the textural properties of the printed products are being evaluated to understand their influence on the properties of the printed product. Table 2 lists some of the studies that tested the influence of the internal structures’ parameters on the textural properties of the products printed.

As seen in Table 2, the internal structure parameters (such as pattern, level, and void fraction) influence the structural and textural properties of printed food. 

The infill level seems to exert the biggest influence on the textural characteristics of the printed products, presenting a positive influence on the weight, hardness, gumminess firmness, Young’s modulus, and fracturability of the printed products and obviously a negative influence on the void fraction [20,65,71,75,85,103,104,105]. Additionally, higher infill levels increase the printing time, processing time, and extrusion rate [75,103,104].

The infill pattern presented a positive influence on the textural properties (hardness) of printed chocolate, potatoes, and yam/potato snacks [85,103,104]. In chocolate, the star and honeycomb patterns resulted in prints with increased hardness and dimensional stability, while with yam/potato snacks, the triangular structures (50 and 80% infill level) provided the greatest hardness [35,85,103]. Nevertheless, even using 100% infill level, the printed structures presented lower hardness levels when compared with casted products [65]. 

Knowing the influence of the parameters on the finished products allows for a more controlled variation of the internal structure to produce customized products. Three-dimensional Printing technology has proven to be capable of customizing the textural properties of the printed products by varying the internal structural design, thus enabling the development of novel sensory characteristics [65,85].

As previously mentioned, one of the major drawbacks of the whole 3D printing process is the time-consuming process of always needing to optimize the printing conditions to the materials used and the printing designs, since, to date, a correlation that can be applied in all cases has not been found. Different materials present different fluid characteristics and since food formulations are composed of different components, that would change the dynamic of the ink, there is always the need to optimize the formulations to achieve a good printing performance [70,77,106]. As for the designs, as they are made more intricate and complex, the tuning of the different variables involved in the printing of the samples is an important requirement [52,76,107]. The development of computer-assisted model simulations that take these factors into consideration and predict the variables’ impact on the printing process could, in theory, fasten the whole process. This point will be further addressed in Section 4.2.

#### 4.1.2. Post-Processing Effect on the Printed Product

Another challenge for 3D printed products is the need for some of these products to go through post-processing, such as baking, frying, and steaming which can cause deformations to the structures and change the products’ characteristics [9,59,82]. Exposure to heat during the cooking process can induce texture modifications, the Maillard reaction, and water evaporation, but also results in changes in the food’s size, color, and nutritional content [3,58,88].

Some studies have attempted to reduce the structural deformations of the printed samples during the post-processing. Kim et al. [82] enhanced the dimensional stability of baked 3D printed cookies by adding xanthan gum to the cookie dough. The addition of xanthan helps to retain the mechanical properties of the samples during the baking process. However, xanthan concentrations higher than 0.5 g/100 g led to an increase in porosity, causing textural changes in terms of hardness and fracturability on the printed cookies [82]. Aside from hydrocolloid addition, alternative post-processing methods such as microwave vacuum drying can help retain and improve the printed structures’ characteristics [108]. By testing various microwave vacuum drying times on mango juice gels, the authors noted that a 4 min microwave drying was enough to retain the structural integrity of the prints [108]. The post-processing also helped with the textural characteristics (hardness and gumminess) of the printed gels, improving their overall acceptability [108].

This point should be further addressed to better understand the post-processing impact on the printed structures and find alternative methodologies with reduced changes to the food products. 

One alternative includes the incorporation of cooking technologies to the printer. Hertafeld et al. [101] incorporated infrareds to cook the food products as they were printed. The authors were able to successfully print multi-material structures out of several pastes (sesame, chicken, shrimp) and cook the layers by infrared heating as they were extruded. This study proves that it is possible to integrate cooking technology into the printer to obtain food products with good resolution and dimensional stability that are cooked as the layers are deposited [101]. This was the first attempt to join the printing and post-processing stages, proving that it is possible to both print and cook the samples at once. Further development of this technique could enable the cooking of the food samples as they are printed, accelerate the fabrication process, and reduce changes to the samples by rendering the post-processing to a minimum.

Three-dimensional Printing can enable the creation of novel healthy products with the possibility of personalizing its profile for specific nutritional necessities [63]. Texture manipulation of 3D Printing products can enable the creation of healthy products with lower contents of salt, oil, and sugar [31,63].

Some healthy products developed with 3D Printing include fiber-enriched snacks using mushrooms and composite flours, cookies fortified with microalgae extracts and insects, orange concentrates enriched with vitamin D, incorporation of probiotics into 3D printed products (using mashed potato and cereal), potato snacks made out of potato by-products and potato snacks with reduced oil content [4,9,80,87,88,103,104,109]. Examples of personalized foods include fruit snacks with specific nutrient profiles which can account for 5 to 10% of the nutritional needs of children age 3–10 and 3D printed vegetable and fish structures with suitable textures for people with dysphagia [5,96].

### 4.2. Model-Based Texture

Computer-aided software (CAD) has so far been mainly applied for 3D structural model design, but it can also be used to simulate the results of the printed products. Guo et al. [106] summarized the requirements for 3D model design, the use of CAD software, object scanning, and others for 3D model building, and the critical parameters setting for 3D model slicing. The authors also presented the possible use of numerical techniques to simulate and predict the 3D food product, helping with the optimization of the model building and the printing parameters. 

Numerical techniques such as the finite element method (FEM), also known as finite element analysis (FEA), can serve as a predictive tool of the mechanical properties and flow performance of fluid materials, and thus, can be used to engineer the desired properties and functionalities of the finished products [106]. FEM can help during the optimization of the printing process because it uses the stress and strain calculations of each element of the model to analyze the behavior of the structures and components under set conditions [110,111].

Vancauwenberghe et al. [102] applied analytical models (linear-elastic model) and FEM to predict the textural properties of hexagonal honeycomb pectin-based structures using Young’s modulus as a texture representative. This study proved that both computational models could predict the decreasing tendency in Young’s modulus with the increased porosity. FEM showed better suitability for low porosity structures in a great variety of patterns, while analytical models showed better predictions for high porosity honeycomb constructs [102]. Some deviations from the modeled geometry were observed on the printed structure, accounting for some of the predictions’ deviations [102]. 

FEM-based software was also used in the prediction of the mechanical properties and the optimization of the printing conditions of pea-protein/alginate mixtures and in the comparison of two 3D printers with different extrusion mechanisms (syringe-based and screw-based) [51,112]. Oyinloye and Yoon [51] applied FEM-based simulations to obtain a geometric model that conferred the printed product, made with pea-protein/alginate mixtures, better structural and thermomechanical properties. By simulating the stress and temperature, in addition to the total deformation of a cylindrical structure, it was possible to infer that these parameters were dependent on the layer thickness, with thicker layers (1 mm) resulting in greater structure deformation [51]. This study demonstrated that these simulations can be used to achieve more complex geometrical structures with predefined properties [51]. In a comparison of two 3D printers with different extrusion mechanisms (syringe-based and screw-based), the simulations suggested that the use of a screw-based printer could be beneficial for the extrusion of multiphase inks, whereas the syringe-based printer was more suitable for simple fluids [112]. The simulation also identified some backflows on the screw-based extruder in the wall/screw flight gap [112]. In the experimental printing, the screw-based printer was unable to print high viscosity materials [112].

Fluid flow numerical simulations can be employed to simulate the materials’ behavior in the nozzle during the extrusion process and thus optimize the formulations and printing parameters based on the materials flow dynamics. By applying fluid flow numerical simulations, Yang et al. [107] were able to improve the 3D Printing of lemon juice gel concentrates. The authors used POLYFLOW, a computational software, to simulate the effect of different materials (different starch types (potato, sweet potato, wheat, and corn) on lemon juice gels) and process parameters on the velocity, shear rate, and pressure field distribution in the cylinder and nozzle during the printing process [106,107,113]. Using a Bird–Carreau model, the authors were able to simulate the effect of the addition of starch to the gels’ fluidity, concluding that the addition of corn starch provided the best characteristics for printing [107]. Changes in the printing parameters exhibit different effects on the pressure distribution in the nozzle, with the nozzle diameter exerting the greatest effect on the pressure with wider diameters resulting in less pressure [107]. Higher volume flow rates lead to increases in the velocity and shear rate and higher material viscosity and volume flow rate result in increased pressure [107].

Much the same was done to evaluate the printability of gels of selected cereal grains and to assess the printing process of power-law fluids [113,114]. The printability of gels of cereal grains (black and brown rice, job’s tear seeds, mung bean, and buckwheat) was predicted by simulating the piston pressure required to extrude the materials using a Bird–Carreau model to describe the gels’ flow behavior [114]. Different gels presented different pressure requirements to be extruded [114]. In the actual printing, the accuracy of the printings varied depending on the material used, with buckwheat and black rice presenting better printing precision, and brown rice exhibiting a lower—but still acceptable—level of printing performance [114]. On the opposite cases of pressure requirements, simulations have shown that mung bean gels required the highest pressures while Job’s tear seeds gels needed the lowest pressures to be extruded, these being the gels that exhibited the lowest accuracy with samples’ deformations, one due to insufficient pressure (mung bean) and the other due to excessive pressure (Job’s tear seeds) [114]. The authors presented the simulation of the materials’ structural mechanics as a tool to evaluate the structural stability and mechanical properties of the materials and prints [114]. Finally, the use of a power-law model to simulate the flow behavior of multi-material formulations (composed of high and low gluten wheat flours, sugar, butter, water, and potato granules) allowed for the prediction of the materials’ effects on the velocity, shear rate, viscosity, and pressure distribution during the printing process [113]. Pressure played a crucial role in the quality of the prints, with low pressure making it difficult to achieve extrusion of the materials through the nozzle, with consequential effects on the samples uniformity; whilst, the application of high pressures resulted in poorly textured and highly deformed printing samples [76,113]. The addition of Potato granules increased the viscosity and the pressure resulting in poor printing accuracy [113]. Better printing precision was achieved by increasing the materials’ temperature which decreased the materials’ viscosity and pressure [113].

The studies presented proved that numerical simulations can be applied in 3D food printing to evaluate the printability of the materials and optimized the printing parameters. However, these simulations have still not been extensively studied and present deviations between the values given by the simulations and the actual values obtained with the printed structures [102,107]. Vancauwenberghe et al. [102] suggested the design of more elaborate geometrical patterns and the use of more complex models to increase the accuracy of the models.

FEM is also used in 4D printing to design, predict, and control the four-dimensional structure’s behavior to different stimuli [60]. Four-dimensional printing will be further addressed in Section 6.

A major limitation to the application of these types of model simulations is the complete lack of specific software that takes into consideration the dynamics of food materials leading to deviations from the actual results [8,106]. The majority of the existing 3D software used for model building, simulation, and slicing was developed to assist in the industrial sector, and thus, more research and development is needed to improve and refine the technique for this specific application field [106].

## 5. Consumers’ Perception and Acceptability of 3D Printed Food Products

As already mentioned throughout this review, 3D Printing presents various advantages that turn it into a technology with immense potential for the food industry. However, one of its major drawbacks could very well be the consumers’ perception of printed foods and their openness to new technologies. The existing studies on consumers’ perception and sensory analysis of printed food products have presented some conflicting results.

In consumers’ perception web surveys, Lupton and Turner [67] reported resistance of the general Australian consumer for food printing, influenced by its appearance, source of food material, visually perceived sensory characteristics, and the perceived natural origin of the printed structures. Most participants showed better receptivity to familiar foods [67]. In another survey, from the same authors, the consumers’ perception of 3D printed meat substitutes produced with lab-grown or insect-based meat was influenced by the participants’ personal feelings and the belief that lab-grown meat was “unnatural” and the use of insects was “disgusting” with the majority of the participants completely rejecting these kinds of products [115]. The limited consumer knowledge of this technology could have influenced the results. 

Brunner et al. [116] also evaluated this topic by studying the effect of 3D printing awareness on the acceptability of 3D printed products and the influence of communication in the consumer attitude change. The authors noticed a positive change of opinion with the increase in information given to the participants, proving that communication can sway the consumers’ receptivity of printed products. An exception was observed in people that already had a preconceived bias towards printing and food neophobia, where the communication was ineffective and even strengthened their opinion [116].

Using a consumer survey comparing 3D printed food with conventional food, Manstan and McSweeney [117] were able to identify three consumer clusters based on their attitude towards 3D Printing. The most representative was the enthusiastic and interested consumer (cluster one), followed by the mildly enthusiastic but moderately interested consumer (cluster two), and the unwilling and not interested consumer (cluster three) [117].

In the sensory perception analysis of printed products, a small scale acceptability study of 3D printed snack bars in a military setting found that commercial snacks were chosen over the printed ones but that the printed snacks’ acceptance grew with repeated consumption [118]. The level of customization in flavor and texture also enhanced the acceptance of the printed snacks [118]. However, as mentioned by the authors, the use of a small sample size (12 person untrained panel) composed of nutritional-aware panelists (soldiers) could limit the strength of the conclusions [118].

In a sensory analysis of printed chocolate with varied infill levels, the chocolate’s appearance was shown to play a crucial role in the preferred choice, with the samples with 100% infill receiving better visual acceptability. Samples with 25% infill were better accepted in terms of texture [71]. The comparison of printed chocolate (100% infill) and cast chocolate resulted in similar preference choices, with the printed chocolate’s preference being justified by its texture (less hard) [71]. Additionally, in consumer surveys, the authors noted that the simple act of displaying the printing process and presenting printed samples could influence the consumers’ opinion of this technology. Contrary to Lupton and Turner [67], the participants did not consider the printed products artificial or unnatural [67]. This discrepancy could be due to the different types of food being presented and the consumers’ awareness of 3D Printing. In Lupton and Turner’s [67] study the participants had limited knowledge of the technology and the biggest rejection was seen on the images with insect-based products and printed meat. On the other hand, Mantihal et al. [71] reported on participants that were mostly aware of the technology and were presented with chocolate samples.

The use of written questionnaires and web surveys on these evaluations could have limited the results and played a role in the outcome of these studies. Nonetheless, some conclusions could be drawn from these consumer surveys such as that 3D Printing is still a relatively unfamiliar technology for the average consumer, but that wider communication and education can influence their attitude towards the 3D printed products [116,117]. It can also be said that a physical introduction, with the showing of the printing process and the tasting of printed products, could be beneficial in attracting the consumers [71,118]. 

The materials’ characteristics, printing parameters, and post-processing can also influence the sensory scores. The printing speed, motor speed, and nozzle diameter influenced the visual sensory scores of rice starch products [68]. The authors noted that a difference of 0.2 mm on the nozzle diameter, resulted in vastly different sensory scores, with a 1.5 mm diameter (at slower printing speeds (<1500 mm/min) and higher motor speeds (180–240 rpm)) showing better overall acceptability, while the 1.7 mm nozzle presented the worst results [68]. The dissimilarities resulted from the poor printing performance at wider nozzle diameters, with the production of structures with over-extrusion and low layer definition [68]. 

Very few articles have attempted to correlate the materials’ properties to the sensory response of the printed product. The first attempt was done by Liu et al. [70], which correlated the viscosity and sensory analysis of egg white protein formulations. The viscosity and sensory scores were shown to have a positive correlation until a viscosity of 1.375 Pa/s was reached from which point the correlation turned negative. A total of 30 trained panelists completed the sensory evaluation and proved the importance of viscosity on the final product [70]. The gum choice (guar gum, xanthan gum, and gelatin) also influenced the textural and taste characteristics of 3D printed pureed carrots formulations [119]. Gelatin addition created more dense, cohesive, and less sweet structures while xanthan gum produces a smoother, sweeter, and with an oily mouth coating purees [119]. The printed and casted puree samples presented similar sensory results [119]. 

Furthermore, the application of alternative post-processing methods such as microwaved vacuum drying also showed an increase in the acceptability of 3D printed mango gels [108]. The printed structures that underwent longer drying times (4 min) presented greater printing accuracy and structural integrity with increased textural properties (hardness and gumminess) [108]. Further research on this topic could help elucidate the effect of the printing and post-processing parameters on the sensory responses to printed products and thus help make the food development process more efficient [119].

Overall, sensory analysis of the printed products showed that some of these were well received by sensory panels for their innovation and customization power, proving that printed foods have the potential to be commercialized [4,9,68]. However, one of the major drawbacks of most of the mentioned studies where consumers’ acceptability was performed was the reduced number of participants, generally with ≤30 participants on the sensory panel, who in most cases were untrained or semi-trained. To produce solid conclusions on consumer preferences, a panel composed of more than 100 untrained subjects is necessary, whereas the use of a trained panel composed of eight to 12 panelists is able to assess, in detail, the foods’ sensory profile [71,120]. From the mentioned studies, only three used trained panelists (Liu et al. [70], Strother et al. [119], and Yang et al. [108] with 30, 12, and 9 trained panelists, respectively, on their evaluation). 

Three-dimensional printing acceptance by the general consumer seems to rely on the people’s awareness of the technology and its benefits. Further publicization of the potential of 3D Printing could help open up the consumers’ receptivity to this new technology.

## 6. Future Trends and Key Limitations 

Three-dimensional Printing has the potential to create tailored food products. However, to reach the consumers, a lot more research needs to be conducted to offset the restrictions and limitations that the technology still presents. This topic will be further addressed below. Nevertheless, these limitations do not seem to hinder the opening of restaurants such as Singularity, scheduled to open sometime in 202X, in Japan, which intends to be a futuristic sushi restaurant that proposes the printing of sushi pieces based on the client’s dietary needs [121].

As mentioned in Section 4.2, a promising tool seems to reside based on the application of numerical techniques to predict the 3D printed products. This tool has the potential to help in food design and quicken the process with fewer resources since it would shorten the traditional trial-and-error stage of the process [110,111]. However, very few studies have approached this technique using food materials and its development is still in the early stages.

The next step in 3D Printing seems to be the introduction of the variable time to the 3D printed structures. Four-dimensional printing technology provides the printed structures with the capacity to adopt different shapes in response to environmental stimuli, such as light, heat, humidity, magnetic field, and pH [60,99,100]. This technique can help in the development of novel textural sensations, with the possible addition of structural changes in the mouth. While still a new technology and not widely used in the food industry, some groups have already tested its applications in pasta that shapeshifts when in contact with water, thus reducing the necessary storage space and shipping costs, and in color-changing potato-based mixtures that spontaneously change color in response to pH stimulus [99,100,122]. Using 3D printing techniques, the structural and color changes were achieved by coupling different materials in one food structure. The shape-shifting of the pasta constructs was achieved by putting ethyl cellulose strips on heterogeneous gelatin films (with a dense top layer and a porous bottom layer) [122]. Once hydrated, the structural changes were based on the geometry and the location of the cellulose strips that folded the pasta in a pre-defined design [122]. The authors presented some examples for its application in the building of the shape-shifting pasta, films that self-wrap caviar, and strips that shortened and twisted in response to the cooking temperature [122]. The multi-material structures of mashed potato-purple sweet potato and anthocyanin-potato starch gels presented spontaneous color-changing properties once in contact with a pH stimulus [99,100]. The mashed potato-purple sweet potato changes were induced by the pH (acidic or alkaline) manipulation of the mashed potatoes [100]. Once printed, the anthocyanins of the sweet potatoes would migrate to the mashed potatoes and change the structures’ color to red (acidic), purple (neutral), or green (alkaline) depending on the pH conditions [100]. The anthocyanin-potato starch gels showed that the structure reacted not only by external stimuli, with the spraying of lemon juice, but also by internal stimuli with the introduction of lemon juice gels to the multi-material structure [99]. In both works, the color changes strengthened with longer storage times [99,100].

3D Printing presents a golden opportunity for the fabrication of food products on-demand specifically for individuals’ needs. However, its adoption by the mainstream depends on overcoming some existing constraints. For that purpose, further and more in-depth research needs to be performed to solve several drawbacks. Table 3 presents some major knowledge gaps in 3D food printing applications.

## 7. Conclusions

Three-dimensional Printing is a tool that has the potential to revolutionize the food industry and promote the age of personalization, allowing the development of products tailored for specific individual needs. Through texture design, the production of healthier food products with lower salt, sugar, and oil contents can be achieved. In 3D Printing, food texture can be designed through multi-material printing and by designing complex internal structures. For now, the existing literature seems to suggest that printed foods present distinct textural properties in comparison with commercial products, for example, in presenting lower hardness levels than their traditional counterparts that make them possible alternatives that are suitable for people with swallowing problems.

Numerical simulations can be used in the evaluation of the printability of materials and printing parameters optimization. Additionally, just like any other novel technology, consumers’ attitudes and awareness towards food that was 3D printed should be taken into consideration and further advertisement and education initiatives should be initiated to highlight the benefits of 3D Printing.

## Figures and Tables

**Table 1:** Some advantages and limitations of using 3D Printing for food printing.

Advantages	Limitations	References
- Personalized nutrition content and development of healthier foods	- Still presents high costs associated	[2,31,35,52,61,65,66,67]
- Freedom to design novel and more complex visual structures and textures	- Low number of compatible materials	
- Food waste reduction	- Still very slow to print	
- Potential to use alternative sources of materials	- Food safety and printer sanitation concerns	
- Can solve problems related to allergies and cross-contamination	- Consumers’ perception	
- Potential to simplify and speed up the manufacturing process	- Limited printing accuracy and surface finishes	
- Decentralized production		
- Reductions in energy and transportation needs		

**Table 2:** Studies correlating the infill parameters with the textural properties of the printed products.

Material	Printer	Structure Pattern	Parameters Studied	Effects on the Printed Products	Reference
Cereal	3D printer Delta 2039	Parallelepiped with internal cubes	- Void number (4, 5, 6, 7, 8, 10, and 12); - Void position	- Pore size and position influence the printed products; - Porosity presents a negative influence on weight, moisture, and water activity; - Porosity influences hardness which decreases with the decrease in the relative density of the printed products; - Increase in dough porosity during baking (exterior to the model) accounts for the decrease in hardness.	[62]
Dough	3D printer	Cube, Cone, and Sphere	- Compressive pressure (300, 400, 500, 600, and 700 kPa); - Needle velocity (3, 6, 9, 12, and 15 mm/s); - Needle diameter (0.25, 0.41, 0.58, 0.84, and 1.19 mm); - Infill levels (10, 30, 50, 70, and 100%)	- Best printing results at 600 kPa, 6 mm/s printing speed, 0.58 mm nozzle diameter, and 50% infill level; - Addition of olive oil and mango powder causes a decrease in hardness but an increase in elasticity and resilience of the printed product; - The printing process leads to further reduction in the hardness, adhesiveness, elasticity, and resilience.	[76]
Dark chocolate	Printer with rotary extrusion (Porimy 1.0)	Cylinder with Star, Hilbert curve, and Honeycomb internal patterns	- Infill levels (5, 30, 60, and 100%); - Infill patterns	- Infill level influences the weight of the printed product, which in turn influences the void on the structures; - Increasing infill percentage causes an increase in the weight of the prints and decrease the void fraction on the structures; - Increasing the infill level also increases the hardness of the products but even a 100% infill presents lower hardness than cast chocolate; - Star and honeycomb patterns provide the most stability and hardness at 60% infill to the printed results.	[35,85]
Brown rice with xanthan gum and guar gum	3D Printer	Cylinder with crossing lines	- Nozzle diameter (0.84, 1.2, and 1.56 mm); - Perimeters (3, 5, and 7); - Infill level (15, 45, and 75%)	- Nozzle diameter and perimeter affect the printed structures’ height and diameter; - All parameters positively impact the weight of the products; - Faster printing speeds can be achieved using wider nozzles at lower infill densities and perimeters, but can cause dimensional deviations; - All tested parameters affect the samples’ texture (hardness and gumminess), with the infill level exerting the biggest effect.	[20]
Yam and Potato	Dual nozzle extrusion printer (Shinnove -D1)	Cylinder with Rectilinear, Wiggle, Triangular, and Honeycomb internal patterns	- Infill Level (20, 50, and 80%); - Infill patterns	- Printed products present a slightly larger dimensional structure than the model; - Infill level influences the porosity, weight, texture, moisture content, hardness, and air-frying processing time; - Higher infill levels decrease the porosity and moisture and increase the weight, hardness, and air-frying processing time (12 min at 20% infill level, 16 min at 50%, and 24 min at 80%); - Infill patterns also influence the hardness of the products, with triangular structures presenting higher hardness at 50 and 80% infill.	[103]
Mashed potatoes	Dual nozzle extrusion printer	Cylinder with Rectilinear, Hilbert curve, and Honeycomb internal patterns	- Infill levels (10, 40, and 70%); - Number of shell perimeters (3, 5, and 7); - Infill patterns	- Infill level, more than the other infill parameters (no effect with the pattern, and limited effect with perimeter), presents a strong influence on weight, void fraction, hardness, gumminess, firmness, and Young’s modulus; - Higher infill levels increase all characteristics except for the void fraction which decreases; - Even at 100% infill level, the printed samples present lower hardness compared to the cast samples.	[65]
Potato	3D Printer	Cylinder with Rectilinear, Cubic, and Honeycomb internal patterns	- Infill patterns; - Infill levels (20, 50, and 70%)	- Infill level and pattern both influence the printing time, extrusion rate, weight, hardness, and fracturability; - Higher infill levels increase all properties tested; - An increase in the infill level and complexity pattern increases the printing time. Longer times are needed to produce honeycomb structures and shorter for rectilinear.	[104]
Dark chocolate	3D Printer (Shinnove -D1)	Rectangular with Rectilinear and Honeycomb internal patterns	- Infill levels (25, 50, and 100%); - Infill patterns	- Infill levels influence the hardness of the samples. Increasing the infill also increases the force necessary to break the samples.	[71]
Wheat dough	3D printer Delta 2040	Cylinder with crossing lines	- Infill levels (10, 15, and 20%); - Layer height (0.3, 0.4, and 0.5 mm)	- Layer height positively influences structures’ diameter and negatively influences the solid matrix fraction and height of the printed snacks. Higher layer height results in a rougher visual aspect of the printed product; - Infill level positively influences the diameter, solid matrix fraction, and hardness of samples; - Cooking of the printed snacks leads to an increase in porosity and weight loss; - Samples printed with a 20% infill level and 0.3 mm of layer height show the highest moisture, hardness, and solid fraction.	[105]
Mashed potatoes and strawberry juice gel	3D Printer	Cylinder with varied internal patterns (triangular, circle, hexagon, and square), a cube with varied layer disposition, and a cube with rectilinear lines	- Mashed potatoes volume ratio (7.44, 20.67, and 41.35%); - Infill levels (40, 60, 80, and 100%); - Infill patterns	- Volume ratio of mashed potatoes influences the hardness and gumminess of the printed products independently of the internal pattern; - Infill levels influence the printing time and rate, hardness, fracturability, and weight of the printed products; - At higher infill levels there is an increase in the weight, printing time and rate, young’s modulus, hardness, firmness, and gumminess, and a decrease in adhesiveness of the printed structures.	[75]

**Table 3:** Some identified knowledge gaps in 3D food printing.

Drawbacks	Comments
Research on the influence of the food materials’ rheological properties and the printing parameters on the printed results. Optimization of the conditions to enhance the printing performance.	Several studies have attempted to establish a correlation between the materials’ rheological properties and the printing parameters on the printing performance (i.e., [6,68,74]). However, by using distinct formulations, the printing conditions present different effects on the printing performance. A definitive correlation between these conditions and the printing results, capable of being applied in most materials used, has yet to be reached, slowing the development of 3D printed products.
Research on the influence of the materials’ properties and printed structures on the sensory response.	A few studies have attempted to test the effects of the materials’ properties (viscosity), products structure (infill level), printing parameters, hydrocolloid addition, and post-processing on the sensory response (see [10,68,71,108,119]). Nonetheless, thus far, the knowledge is still limited and scattered. Further research on the influence of the mentioned parameters on the sensory response would benefit the development of printed food products with good consumer acceptability.
Applicability of CAD software for modeling, slicing, and as a predictive tool for the texture of designed structures [106].	Computer-aided software (CAD) software is used during the modeling and slicing stages of the printing process, however, most of the software was developed for the printing of thermoplastic materials and does not take into account the properties of food materials [8,106]. The same happens with finite element method (FEM)-based software that is being used to predict the mechanical and flow properties of the materials [102,107]. The fact that the existing software does not consider the food’s properties will create dimensional discrepancies between the virtual model and predicted structures and the real printed products [8].
Development of personalized software for specific individual needs considering parameters such as age, occupation, nutritional profile [106].	People have different nutritional needs according to their specific age, occupation, activity level, etc., and thus, each person will present an individual nutritional profile. The development of personalized software that considers these factors would enable the production of tailored food products for specific groups of people [106].
Development of hygiene protocols for the printer parts Food safety research.	At least one study has shown microbial growth on the printed products, which suggests the possibility of contamination during the printing process [66]. Other possible hazard risks include the fragmentation of printer parts or the production of health-hazardous compounds [24]. Thus, as key factors in the food industry, sanitation protocols and further studies on the safety of 3D printed food should be performed.
Printing of more sustainable traditional food alternatives.	Some alternative food material sources have been successfully printed (algae, mushrooms, insects) but their use is still limited [9,58,88]. In the specific case of the printing of meat, it could be served as an alternative for people that follow a vegetarian diet. As the meat is printed with alternative sources or cell cultures, there is no need to sacrifice live animals, and thus it can be consumed without the conflicted views associated with animal consumption [57].
Incorporation of other established technologies like microencapsulation, electrospinning, and infrareds [57].	Microencapsulation and electrospinning can potentiate the introduction of functional ingredients to the food formulations [12,57]. Incorporating cooking technologies to the printer would accelerate the printing process [101].
